# Repertory of the Back

**Published:** 1898-03

**Authors:** E. H. Wilsey

**Affiliations:** Parkersburg, W. Va.


### REPERTORY OF THE BACK.

E. H. Wilsey, M. D., Parkersburg, W. Va.

(Continued from page 35.)

**Shirt**, sensation on skin of back as if he wore a stout woolen shirt, Fer-sul.

**Shivering**, in back, Cepa., Dig.

— on back with tenesmus and small loose stools, preceded by terrible soreness in intestines, Thromb.

— intense fever, commencing with chills running down back like streams of cold water, causing shivering and chattering of teeth, Variol.

— from the feet up the back, extending into the arms at 6 P. M. for one-half hour, Sul.

— in back in A. M., with frequent yawning and inclination to sleep, Graph.

— running down back, Chel., Variol.

— and shuddering in the back chest and epigastrium, Mez.

**Shiver**, feverish shiver every A. M. at 9 o'clock, with some nausea without any subsequent heat, Mag-c.

— pinching and cutting below the navel with shivers over the back, then heat in head and urging to stool at noon, Mag-m.

— over the back with stitches in the head, Mang.

— all over back, with red cheeks and cold hands, Euphorb.

**Shock**, an undulating shock in back, causing him to start with a fright, Stann.

**Shooting**, constant pain in back shooting through body to both sides and along spine to occiput, and even to temples at times, worse by walking and stooping, *Coccul.*

— pain down right side of back, Fer-sul.

— pain goes from right to left side of back and shoots down legs, Lob.

— sharp pains up back with hemorrhoids, generally blind or aching, with lameness, Æscul.

— small furuncle on back, with shooting pains when touched, Mur-ac.

— from left side of back through to chest when taking a deep breath, Mez.

— itching pain in middle of back, ceasing when rubbed, Mang.

— violent pain in back between hips, Sil.

— weakness and pain in small of back, and sharp shooting from lumbar vertebra to crest of ilium 6 P. M. on right side, Chrom-ac.

— burning and still more shooting in the back, seemingly in the marrow, Mag-m.

— also in back, while standing very violent, Zinc.

— and stitches down from back to hip, *Kali-c.*

— several paroxysms daily of half an hour duration, first a griping and clutching in back, when it comes like a shooting into the side, Lyco.

— and tearing in the back when moving, especially at night, Nit-ac.

— violently on left side of back so that she dare not move, but movement brought relief, Mur-ac.

— frequent in the back after diarrhœa with colic, Calc-ars.

— through small of back, Lyss.

— and stitches in back at times, on the right side of chest in the evening, also at night, disturbing sleep, Nat-c.

— deep in the cardiac region extending to axilla and back, with shooting in the thigh, extending to the heel when sitting, ceasing on rising up in evening, Mur-ac.

**Shooting,** burning fine shooting on a small spot in middle of back, Stann.

— and burning in the back in the morning, ceasing after rising, but the back remains sensitive and as if bruised, Nat-c.

— and pressive tearing in the back near the scapula, Kali-c.

— violently while lying on his back, Kali-c.

— in chest and muscles of back, Sul.

— fine in the back outward, Stann.

— from back to stomach pit, Rhod.

— in back toward sacrum when sitting, Lyc.

— excoriating shooting, in the right half of the back as from needles, Plat.

— from small of back into left hip bone at 3 P. M., Chrom-ac.

**Short,** muscles of the back feel too short when bending forward, Agari.

— pain in small of back, tensive as if everything were too short, and violently on stooping, *Sul.*

— drawing in back as if it were too short to allow stooping without pain and difficulty in keeping upright, Calabar.

**Shoulder** and back pains, Stram.

**Shudder** in the back, in evening after lying down, without subsequent heat, Nat-c.

— over the back and arms, Mez.

— heat in the evening with a thrill of cold and shudder over the back without thirst, Nat-m.

— when startled at night shudder in back, Carbo-v.

— in back especially while sitting, Nat-m.

**Shuddering** over back, extending into feet and arms, Calc-ars.

— chill over back, Phos.

— in back, Carbo-v.

— over single parts of back, Ars-hyd.

**Shuddering** and feverish chill in back, Guaic.

**Sickness,** bruised pain feeling as if a severe sickness had seized him, Myrica.

**Side,** pains which are excited or worse by lying on side, are better by lying on back, *Puls.*

**Sinking** sensation going from back to heart, Lyss.

**Sitting,** patient cannot sit at all, they are very much worse by sitting, particularly backache, Zinc.

— scarcely able to rise or stoop after sitting, *Puls.*, *Æscul.*

— stiffness in back on sitting down in morning, Iris-v.

— stitches in small of back when sitting, Kali-iod, *Nat-c.*, Anac., Ambra.

— a drawing down back when sitting, disappearing during motion, Bry.

— intermitting severe stitches always in one spot in middle of back when sitting, Euphorb.

— stitches in small of back when sitting, stooping, or walking, better by pressure and lying down, Ruta.

— drawing in back while sitting down in evening, Tereb., Carbo-v.

— stiffness in small of back after sitting, Ambra.

— pain in small of back, worse when sitting still or when lying down, better by lying on something hard or from exercise, *Rhus-t.*

— constrictive pain in dorsal muscles while sitting and better by bending back, worse by bending forward, Rhus-t.

— pain in small of back, worse when sitting, *Cimex-lec.*, Oleum-an.

— bruised pain in small of back while sitting, in P. M., Hyos.

— pain in small of back when sitting or when in *the* act of sitting down, diminished by continuous walking, Zinc.

— turning causes sudden stitches in back, with dull pains while sitting, *Nux-v.*

— pain in back more while sitting, Zinc.

— pain in small of back while sitting, Prunus-sp.

**Sitting**, pain violent in small of back while sitting not allowing to straighten, *Lyc.*

— violent pain in small of back while sitting and lying, better by motion, Agari.

— violent pain in left loin with stiffness of back when rising from sitting, and prevents straightening the body; while sitting he feels no pain and can turn to every side without pain, Agari.

— violent pain in back while sitting for a time, Phos.

— pain in back while sitting, better by walking or lying, *Cobalt.*

— intense pain in back while sitting, worse when lying, not while walking or performing manual labor, Nat-m.

— drawing pain when sitting, Thuya.

— frequent rising of heat from the back into the head, with redness of the face in the P. M. while sitting, Phos.

**Sleep**, at night in sleep he always lies on his back, Lyco.

— shivering in the back in A. M., with frequent yawning and inclination to sleep, Graph.

— restless sleep, can only lie on his back, Dig.

— at night first sweat on back causing him to wake up at 4 o'clock; then dry internal heat with uncomfortableness so that he cannot go to sleep again, Petrol.

— weakness in small of back as if it were going to sleep, while rising from a seat, Phos.

— back aching him awakens him from sleep, *Hydrast.*

— could not sleep during whole menstruation on account of tearing in back, chills, and heat, with thirst and painful contractions of chest, *Sepia.*

— nocturnal pains in small of back, which always awake her from sleep, *Amm-m.*

— pressing, drawing pain in back and loins, which makes turning very difficult, it awoke him from sleep, *Bry.*

— constant pain in small of back, worse by motion and stooping, disturbing sleep at night, Sul.

**Sleep,** back so painful cannot sleep; passes no urine, Arenea-dia.
— cannot sleep on account of pain in small of back and hips, better by motion, Sinapis-nig. and Alba.
— carbuncle on left side of back very large, on scapula surrounded by small pustules, scanty and offensive pus, great pain depriving him of sleep, weakness and prostration, *Hepar*.
— pain in small of back renewed by falling to sleep, Mag-m.
— pain in cervical muscles at night as if head had been held in an uncomfortable position, even felt during sleep, *Zinc*.
— pinching in stomach which contracts the chest with grasping together of back, awaking her on moving, Con.
**Sleeping,** nearly always on his back, one hand under his occiput and the other above the head, Coloc.
— a jerk in back at night while sleeping, Amm-c.
— weakness in back and limbs with sleeping, Gels.
— heaviness in back in morning as if he had lain in a wrong position, with weariness as if he had not slept enough, Sul., *Sep*.
**Small,** drawing pain in small of back, Carduus.
— pain in small of back, *Sepia*, *Bar-c.*, *Coloc.*, Sul-ac., Ran-s., Secal., Can-sat.
— pain in small of back and loins, Carbon-sul.
— pain in back and sacrum, *Cap*.
— pain starts from small of back, spinal bones affected, *Calc-c*.
— pain in small of back, worse by motion, *Bry*.
— pain in small of back and between lumbar vertebra, false ribs and ilium, Croton.
**Smarting** in back from grief, *Naja*.
— burning pain in small of back, Zinc.
**Sneezing,** after falling from a height upon back acute pain in lower part and in right side of back, especially when laughing, sneezing, or taking a long breath, *Con.*

**Sneezing,** pain in right side of back, followed by violent sneezing, Anagallis.

**Sore** pain and aching across small of back, *Lact-ac.*

— pain in small of back unchanged by motion, Brom.

— a pain commencing in right side of back, then going down to os ischium, very sore to touch, Lobelia-c.

— pain in back as if sore or bruised, Sul-ac.

— arms and back tired and sore all over, Sarrac.

— aching like a sore eruption on back, Bry.

— lame feeling in small of back, *Lept.*

— contraction in back with sensation of coldness, griping and sore feeling, Con.

— denuded sensation in left side when sitting with burning intermittent sticking, Plat.

— extremely sore back as if beaten, in evening, Lyss.

**Soreness** of back, *Arn.*, *Benz-ac.*, Agari., *Carbol-ac.*, Cornus., Pod., *Rhus.*, *Sang.*, Sarrac., Lyss., Ars-iod., *Tereb.*, Ol-jec., *Apis.*, Nat-s., *Caust.*, *Lact-ac.*

— and backache with kidney affection, *Tereb.*

— from back to chest, Oleum-jec.

— intolerable aching soreness in small of back as if beaten, Eup-per.

— drawing pressure in first two dorsal vertebra, Staph.

— smarting and weakness of back, Podo.

— in small of back on rising with rumbling in abdomen, Fer.

— of muscles of back and limbs, *Carbol-ac.*

— of back and neck as if beaten, Ars-iod.

— down muscles of back, *Sang-c.*

— sensation of soreness and weariness in small of back, Cimex-lec.

— in middle of back, Thuya.

— in intestines with small loose stools, shivering along back with tenesmus preceded by soreness, Trombid.

**Soreness,** great of back, Arn.
— in small of back when touched, Colchi.
**Spasms** in back with severe pressing and drawing, Con.
**Spasmodic** drawing in small of back, compelling him to lie still, *Sil.*
— stitches in paroxysms in middle of the back which render motion impossible for some minutes, Lyc.
— pain in back, Pallad.
— pain in dorsal vertebra while lying in bed in morning, Euphorb.
— pain in anterior side of chest and in back awakes him from sleep, Nit-ac.
— pain in small of back would not allow him to arise, *Sul.*
**Spina bifida,** *Psor.*
**Spleen,** pain and aching in posterior aspect of spleen, *Lob-c.*
**Split,** great weakness in back as if it would split and fall apart, *Lyss.*
**Sponge,** hot near back causes him to wince, *Phos.*
**Spot,** burning in a small spot on back, *Nit-ac.*
— tearing in back in a small spot, Caust.
— small painful spot low down in back, *Lach.*
— on back, pains when touched, Stram.
— pain in back with sensitiveness of affected spot by leaning upon it, Plumb.
— burning pain in a spot about small of back, Phos-ac.
— back pains in spots when sitting, Thuya.
**Sprain,** pain as from a sprain in both sides of back, Calc-c.
— as from a pain in left side of back, Con.
— pain in shoulder and back with stiffness as from a sprain, *Rhus-t.*
— pain in right side of back as if hand pressed it or as from a sprain, Oleand.
— pain as from a sprain in region of small of back on left side, Agar.
— pain as from a sprain in left side of back and neck, *Con.*

**Sprained,** back feels as if sprained in P. M. and evening in bed, *Sepia.*

— pain in back as if from a false step, Sul.

— pain in abdomen and back during fever, *Lach.*

— pain in back and in scapula extending into chest, two or three times per day, arresting the breath, Petrol.

— pain in back as if he had stooped too long, *Mur-ac.*

**Squeezing** pains in back tensive, Arg-n.

— in back as if with effort to stool, Berb.

**Stabbing,** in back through to umbilical region, *Ver-v.*

**Stand,** back very weak, unable to stand, *Physos.*

**Standing,** stitches through small of back, with drawing through lumbar vertebra while standing, *Con.*

— pain in small of back, worse while standing or walking, *Kali-c.*

— a vivid pain in small of back like drawing and pressing, occasionally tearing, only perceptible while standing, Phos-ac.

— pain in small of back, better by standing or walking, but severe when rising from seat, *Arg-n.*

— tensive pain on standing erect, Ign.

— causes back to ache, *Sul.*

— weakness in back, can hardly stand alone, Sul-ac.

— feels bruised on small of back, especially while standing, Agari.

— violent stitch in middle of back while standing, Zinc.

— shooting in back, while standing very violent, Zinc.

— stitches like bearing on left side of back upward while standing, Stann.

— violent constant stitch in dorsal vertebra when standing, Nit-a.

— lower part of back lame when walking or standing, Zing.

— if she stooped lower part of back felt stiff, it was with difficulty she could stand up; tearing when sitting, worse from standing, better in P. M., *Berb.*

**Standing,** backache causes nausea and faint feeling while standing, *Sep.*

**Step,** sharp drawing across small of back, sensitive to every step, *Carbo-a.*

**Sternum**, drawing pain in sternum extending to umbilicus and back, Raphn.

**Stick,** sensation as if a round stick pressed forward and upward from about last dorsal vertebra into stomach, and awaking at 2 A. M., Nat-p.

**Sticking** and cutting through back and abdomen, Canth.

— in back, in general, Cup., Sul., Spig., Pæonia, Rhus-t., Graph., Iod., Ver-a., Droser., Psor., Nat-ph., Canth., Jambos., Merc., Lyc., *Puls.*, *Nit-ac.*

— lightning-like in tabes dorsalis, *Nit-ac.*

— burning itching in small of back, Jambos.

— extending toward chest when coughing, Psor.

— in lumbar muscles of back, Drosera.

— burning with tension in small of back, Ver-a.

— upon breathing in small of back, Merc.

— in small of back, Graph., Iod.

— in back while stooping in evening, Rhus-t.

— periodically in places in back, better by scratching, Pæonia.

— transversely across small of back, Cup.

— in back opposite heart, Spig.

— at every breath on walking, itching, *Sul.*

— drawing pain in small of back, Stront.

— pain in back and shoulders, with tension, Ver-a.

— drawing pain in back, Lyc.

— pain in back and across chest, *Puls.*

— fine sticking pain in back, *Puls.*

— lightning-like pains of a sticking character, in back, *Nit-ac.*

**Stiff,** pain in small of back as if stiff, *Nit-ac.*

— lower part of back feels stiff and heavy, Zing.

**Stiff,** all parts of back feel stiff as if he had been injured or over-lifting, *Prunus-s.*

— and lame, weakness in small of back, Diosc.

— in back she can't stoop, Kali-c.

— in back and sides as after taking cold, Sul.

— if she stooped lower part of back felt stiff, it was with difficulty she could stand up; tearing when sitting, worse when standing, better in P. M., *Berb.*

— back very stiff every morning and in damp weather, *Phyto.*

— back very stiff and not very painful, Stram.

— as if beaten in back, disabled and stiff, Berb.

**Stiffness** of back (undefined), Aco., Amb., Copai., Sul., Sul-ac., *Cup-ars.*, *Sep.*, *Guaic.*, *Nit-ac.*, *Bar-c.*, Petrol., *Berb.*, *Rhus-t.*, Sil., *Coccul.*, Sal-ac., Lyc., *Apis.*, Nat-m., Ars., Lauroc., Zinc., *Prunus-sp.*, Diosc., Kali-c., *Phyto.*, Stram., Bovis., Dig., Ananth., Bapt., Aur-mur., *Carbo-veg.*, *Helon.*, Med., Phos., *Puls.*, Fer-iod., Cup-m., Chelid., Chromic-a., Æsc-h., Cup-s., *Caust.*, *Calc.*, Bry., Ang., Agari., Ars-h., *Cic.*, *Ign.*

— of trapezius muscles before nervous headache, *Ign.*

— in small of back in the evening, especially while sitting, which allows neither to arise nor bend backward, Bar-c.

— of back, can hardly rise from a chair, *Bar-c.*

— in small of back and neck, *Sep.*, *Rhus-t.*

— and chilliness in nape of neck and all down back, Sil.

— of lower part of back, *Sep.*

— of cervical muscles and great weakness of back, *Coccul.*

— in back at night on waking, Sal-ac.

— in back after horseback riding, stooping or walking, Lyc.

— in back and sacrum, *Apis.*

— and rigidity in the nape and across the upper part of back, Nat-m.

— painful in back all day, Ars.

— in left side of neck and small of back, Lauroc.

— and drawing in the back, Petrol.

**Stiffness** with backache after stooping, Bov.
— and paralysis of back and sacrum, Kali-c.
— from scapula down back, Lyc.
— in back and sides of neck with thrust-like pain, Dig.
— and heavy feeling in lower part of back, Zing.
— of back as from an injury, Prunus-sp.
— tetanic stiffness of the back and legs, with drawing pains in thighs and calves, *Cic.*
— and pain in small of back, *Kali-c.*
— of back, painful mornings on rising, Carbo-v.
— and pain in small of back at night, *Lyc.*
— painful in back, Ver-alb.
— and pain in back on moving, Cup-s.
— in muscles in middle of back with pain, Sal-ac.
— and pain in upper dorsal region, especially on movement, worse at night, Zinc.
— painful in small of back, *Mancin.*
— rheumatic of whole left side of back from nape down to sacrum, with intolerable pain on least motion, not noticed on touch or during rest, *Guaic.*
— after sitting, Sul., Amb.
— as after taking cold in back and in sides, Sul.
— in morning, better during day on motion, Sul-ac.
— of back, better by walking, Copaiva.
— and lameness of back, worse from motion, better during rest, returns after sitting a while, *Cup-ars.*
— and great uneasiness in small of back and coccyx in evening, Petrol.
— excessive of one side of the neck, extending to small of back, worse by moving, *Guaic.*
— cramp-like of back and whole body, *Nit-ac.*

**Stinging** in back, *Apis.*, *Cham.*, Rumex.
— piercing twitching in the back and sacrum, taking his breath, Caust.
— in back as of minute insects, Chlor.

**Stinging** pain in small of back, *Aur-m.*
— pains in small of back, with weak sensations, Merc.
— nettle-like pains over back where it touched the bed, sixty hours after delivery, *Kali-c.*
— periodical spasmodic pain in back, *Sul.*
**Stitches** in back in general, Kali-c., *Bry.*, Staph., Sul., *Nat-c.*, Spig., Stann., *Con.*, Nit-ac., *Sars.*, Alum., *Calc-c.*, *Nat-m.*, *Hepar.*, Mang., Euphorb., Kali-iod., Lach., Zinc., Mez., Verbas., *Ign.*, Ind., Mag-c., Caust., *Puls.*, *Hyper.*, Niccol., *Guaic.*, Dig., Apis., Lyc., Ruta., Secale., Carbo-an., *Sep.*, Plumb., Psor., *Dulc.*, *Kali-c.*, Asaf., *Chin.*, *Mag-m.*, Anac., Cornus., *Kreos.*, Berb., Chel., Form., Colch., Coccul., Callad., Chen-v., Cinnab., *Nux-v.*, *Camph.*, Merc-s., Cepa., Agnus.
— and pain in small of back and both hips and in left chest at night, *Lyc.*
— turning causes sudden stitches in back, dull pains while sitting, *Nux-v.*
— pain in small of back and abdomen, with stitches in left side and drawing pains in lower extremities, Zinc.
— painful in left side of back when sitting, Mur-ac.
— pain in chest, stitches in left side, under true ribs more toward back, Guaic.
— in small of back pain like rheumatism, Iod.
— small and violent between scapula, Sarsa.
— fine from the back to region of ribs, Alum.
— severe in middle of back from time to time, Alum.
— in right hypochondrium extend toward small of back, *Calc-c.*
— at night when lying on his back he starts up and feels a stitch in right side of chest, Nit-ac.
— beaten, bruised feeling in back with stitches, *Nat-m.*
— sharp transversely through small of back, close above hips, Nat-m.
— intermittent in back above right hip, Carbo-a.

**Stitches** in back and renal region, Hepar.
— stitch on lowest rib close to back, Mag-m.
— in the head with shivering over the back, Mang.
— intermitting severe stitches always in one spot in the middle of the back when sitting, Euphorb.
— in small of back when sitting, Kali-iod.
— in back from below upward, Lach.
— in right side of back extending through to chest, Kali-c.
— shooting in back through into chest, *Bry.*
— in back and small of back, Bry.
— violent upward in back, Staph.
— itching in back, Sul.
— in back sometimes as far as the chest (left side) evening and night, Nat-c.
— in small of back after sitting a while, *Nat-c.*
— an occasional stitch from small of back through left side of abdomen toward chest, Kali-c.
— in back when breathing, Spig.
— in back and small of back into limbs, Stann.
— in small of back with drawing through lumbar vertebra while standing, *Con.*
— and shooting down from back into hips, *Kali-c.*
— in back at every breath, Sul.
— in back and then backache, Caust.
— in muscles of back, Asaf.
— in left side of back, *China.*
— repeated in the back above the renal region, Lyc.
— tearing and burning in small of back, *Mag-m.*
— blunt in small of back, Anac.
— from the depths of chest going out at the back, Caust.
— in small of back, *Puls.*, Ign., Niccol., *Hyper.*
— in left side below true ribs rather toward back, *Guaic.*
— lacerating and sharp in small of back, Dig.
— burning in upper part of small of back, Apis.
— in back toward small of back when sitting, Lyc.

**Stitches** in back through into chest from least motion, *Sarsa.*
— in back when coughing, *Sepia.*
— in small of back and between scapula, Plumb.
— backache when walking, with stitches in sternum, *Psor.*
— severe rheumatic in back and arms after taking cold while sweating, worse at night and while resting, better from motion, slight fever, much thirst, *Dulc.*
— a sharp twitching stitch in left side of back and at same time in left thigh, Stann.
— like bearing on left side of back upward while standing, Stann.
— violent in middle of back while standing, Zinc.
— in middle between right loin and spine intermittent and deep, sharp knife-like stitches quite internal in intestines, Verb.
— dull into back, Mag-c.
— lancinating as from a sharp knife in back through loins extending to legs, *Ign.*
— in small of back, better by an evacuation, Indigo.

**Stitching** pain in small of back so severe upon motion that he gets upon arms and knees in order to obtain relief, pains unbearable in any other position, *Coloc.*
— pain in small of back, Sul., Zinc.
— drawing, sometimes stitching, pain in small of back and lower down in abdomen, Zing.
— pain in small of back and legs on being touched, Merc.
— pain with unsteadiness of back, knees, and feet, Merc.

**Stomach,** pain from stomach frequently moves to back, *Kali-c.*
— some pain in back opposite stomach, comes on an hour or so after meals, causing difficulty in speaking, Sul.
— pain in back opposite stomach, with dyspeptic pain, better by eating a few mouthfuls of food at 11 A. M., Phos.
— pain in back starts from post wall of stomach and spreads under short ribs of left side, obliging patient to bend forward, *Nux-v.*

**Stomach**, afternoon a burning pain in back as if in post walls of stomach, Lob-i.

— contractive pain in stomach with sensation of coldness in it and in the back, awaking her from sleep in the morning, Con.

— sharp pain extending from chest and stomach through to back, between shoulders, worse on right side, Codein.

— sensation as if a round stick pressed forward and upward from about last dorsal vertebra into stomach, awaking at 2 A. M., Nat-phos.

— stitch striking from back to pit of stomach in P. M., when sitting, Niccol.

— shooting pain from back to pit of stomach, Rhod.

— burning in lower half of body from small of back to pit of stomach downward, Phos-ac.

**Stool**, backache during stool, Apis, *Ars.*, Colch., *Nux-v.*, *Cub.* (and before).

— chill in back during stool, *Tromb.*

— chill in back after stool, *Puls.*

— backache with desire for stool, *Plat.*

— backache after hard stool, *Ferr.*

— pain in back before stool, Bapt., Cic., Nux-v., Puls.

— pain in back after stool, Cap., Merc-v.

— pain in small of back, better after stool, Ox-ac.

— pain in back during stool, *Dulc.*, *Puls.*

— pain in back, worse during stool and continuing afterward, Podo., *Cap.*

— pain in small of back with hard stool and colic, as if intestines would burst, *Lyco.*

— labor-like pains in small of back and sacral region, urging to urinate and ineffectual desire to stool, *Kreos.*

— coldness in back before stool, Ars.

— flashes up back with stool, Podo.

**Stool,** chilliness in small of back after stool, Puls.
— squeezing in back with effort to stool, Berb.
— shivering along back with tenesmus and small loose stools, preceded with terrible soreness in intestines, Tromb.
— throbbing in small of back during and after stool, Alum.
— pinching and cutting below the navel, with shivers over the back, then heat in the head and urging to stool at noon, Mag-m.
— pain in small of back most severe after stool, Tab.
— pain in small of back during soft stool, Niccol.
**Stoops** or bends forward when walking or sitting, does not walk erect, *Sul.*
— impossible for him to stoop or lie upon back, Tereb.
— if she stoops lower part of back feels stiff, it was with difficulty she could stand up, tearing when sitting, worse on standing, better P. M., *Berb.*
**Stooping,** stiffness in back after horseback riding, walking or stooping in lumbar region, Lyc.
— sensation as if vertebræ were being torn apart when stooping forward and then bending backward again, *Chel.*
— stitches in small of back when sitting, stooping, or walking, better from pressure and when lying down, Ruta.
— sensation as if bruised in back and sacrum, worse from stooping and when touched, Stront.
— sensation in small of back and hips while stooping as from a girdle, Gentiana.
— backache worse from walking, also drinking coffee and when stooping, *Cham.*
— small of back as if broken when stooping, worse with tension, Clem.
— while stooping, pain in back, Sul., Cap., Amm-c., Nitrum.
— while sitting, small of back aches as after long stooping, *Rhus-t.*
— after stooping with difficulty he could become erect, Lach.

**Stooping,** darting across right side of small of back, worse on stooping, passing away gradually, Lact-ac.

— pressure in middle of left side of back as from much stooping, Mur-ac.

— pain in left side of back as after long stooping, Bism.

— is obliged to walk stooping and gets painful stitches in back by accidentally kicking foot against something, *Sep.*

— pain in small of back after stooping, *Sil.*

— pain in small of back violent only on stooping, tensive as if everything were too short, *Sul.*

— bruised pain in small of back, worse when stooping, also when walking, *Meny.*

— aching pain in small of back when stooping, Jug-cin.

— pain in small of back on turning over at night and stooping, Zinc.

— on rising from stooping pain in small of back, Phos-ac., *Phos.*, Amm-m.

— pain in back as after long stooping, Graph., Agari., Bry., *Dulc.*

— pain in small of back after or from long stooping, *Puls.*

— after long stooping pain in back as if bruised, mostly while straightening, *Nat-m.*

**Stove,** a thrill of chills creeps up the back, better by warmth of stove in evening, Sul.

— burning on skin of whole back as if he was sitting by a hot stove and sweat on face with moderate heat, Dulc.

**Straight,** great weakness of muscles of back, he can hardly sit straight, Agari.

**Straightened,** body and legs straight, head drawn back, if lying on side is straightened out upon back as quick as lightning, *Cic.*

— could not straighten back, *Calc-ars.*

— severe backache as if bruised, cannot straighten out, *Psor.*

— after long stooping, pain in back, as if bruised, mostly while straightening, *Nat-m.*

**Strain**, pain in back as from a strain on lifting, in scapula after continuous writing with back bent, Mur-ac.

— pain as from a strain in back, Kali-c.

— crick in back caused by strain in sudden lifting, Sep.

— and drawing into the back extending into the arms, in single jerks and terminating in a stitch when sitting or lying down, Nat-c.

— pain in back in morning, Dros.

— pain *streaking* up and down back, *Phyto.*

**Stretch**, tension in back compelling him to stretch and extend limbs, Nat-m.

**Stretching** in back with chilliness at night, Phos.

**Strength**, loss of strength in small of back, *Ars.*

**Sudden**, sharp, sudden pain in left side at tenth rib in P. M., Diosc.

**Supper**, repeated cramp-like twitching in the whole back after supper, with pain in back, and then also in right side of abdomen, Sul.

**Support**, lameness of back, cannot sit up without support, Diosc.

— she feels as if the muscles of the back were not strong enough to support the body, which always tends to fall forward, Amm-c.

**Suppurate**, on back and thighs painless dark red pustules which do not suppurate, Chima-u.

**Swallowing**, pain between shoulders on swallowing food, *Rhus-t.*

— pain in back on swallowing, *Kali-c.*

**Sweat** on back after meals, Card-m.

— on back in evening, Anac.

— on back during menses, *Kreos.*

— on back from least motion, *China.*

— cold sweat on back from least motion, Tart-em.

— on back cold at night, *Sep.*

— on back during effort to stool, *Kali-b.*

**Sweat** on back, Stram., *Petrol.*, Hyos.
— on back generally worse, Dulc.
— chilliness in back and as if sweat would break out, Camph.
— injury to back or checked sweat, *Aco.*
— all over, with severe thirst after the sweat and after a shaking ague, and great coldness in bed as if she lay in ice for two hours, with drawing in limbs and back, Lyc.
— profuse on head and back every second or fourth evening three-quarters of an hour, Mur-ac.
— especially about midnight on back, Hepar.
— on back with sensitiveness to cold air in the morning, Chim-m.
— severe rheumatic stitches in back and arms after taking cold while sweating, worse at night and while resting, better from motion, slight fever, much thirst, *Dulc.*
**Swollen,** from cervical vertebra down to lumbar region, right side of back, three or four inches in width, much swollen, elastic, dull pains, intolerable after exertion, has to lie down in a kind of tetanic state, *Lach.*
**Sympathetic,** dorsal symptoms painful, perhaps sympathetic with the uterine sphere, Zizia.
**Tearing,** burning, drawing, lacerated or bruised pain in back, *Nux-v.*
— pains, small of back seems as if it would break, *Ham.*
— pain in nape of neck, extending thence gradually half way down back, *Rhod.*
— stitching pain in small of back in evening before going to sleep, in bed, Alum.
— pains in back at night, Phos-ac.
— pressive pain on left side beside the hip, extending to back, Carbo-v.
— violent pain in back and limbs, worse from least touch, *Chel.*
— in back in general, Ant-cr, *Mag-m.*, Carbo-v., Caust., Alum., Sil., Mez., Sul., Croc., Cup., Petrol., Mag-c.,

*Ars.*, Nit-ac., Nat-s., *Berb.*, Stann., Sep., *Phos.*, *Canth.*, Brom., Cham., Cinnab., *China.*, *Cap.*, *Nux-v.*, Agar., *Chel.*, *Calc-ph.*

**Tearing**, back after eating, afterward to abdomen, Cham.
— in back during chill, *Cap.*
— from back into limbs with paralysis, *Phos.*
— in back during menses, Sepia.
— in back during profuse menses, Agari.
— in back, worse from least touch, *Chel.*
— in back while walking, *Canth.*
— in back especially in morning, *Canth.*
— in small of back, worse by breathing, Croc.
— and burning in small of back, Cup.
— in back and between scapula, so could not move, Petrol.
— violently in nape of neck and twitching, proceeding gradually down the back, and there eventually ceasing, Mag-c.
— greatly exhausted from sexual excess, pulling and tearing in back and legs, *Ars.*
— and shooting in back and chest when moving, especially at night, Nit-ac.
— from back down with feeling in bones as if dogs were gnawing them, Nat-s.
— when sitting, worse when standing, better in P. M., the lower part of back, *Berb.*
— in back in evening in bed, and in knees and legs, Sul.
— stitch-like tearing upward in back when standing (left side), Stann.
— in back from morning till night, Ant-cr.
— and burning stitches in small of back, *Mag-m.*
— in lower part of back beside spine, Carbo-v.
— in small spot in back, Caust.
— in vertebræ of back between the scapula, extending into right, then into left scapula, Caust.
— severe in arms and hands, extending into back, Caust.
— severe or pecking pressure in the back, with a chill, later passing over into a dull pressure, headache with heat in head, Sil.

**Tearing,** in left side of the back in the morning, Mez.

**Tear,** great wearisome pains in thighs as if the tendons would tear off, alternating with pains in small of back, she knows not what to do for the pains third day of menses, Amm-c.

**Tension,** painful on right side of nape and in small of back, slight in left side of nape, Thuya.

— between shoulders and down back, Mag-m.

— in back before dinner, Niccol.

— in back, Rheum.

— in back obliging him to stretch, Nat-m.

— in back as if compressed in a vise, in sitting, passing off through motion, Amm-m.

**Tensive** pain in back, Con.

— pain in back after dinner and at night, sometimes only when sitting bent forward, and then it ceases on stretching himself, Nat-c.

— frequent waking after midnight till toward morning, he lies on his back, with open mouth, dry tongue, tensive pain, and heaviness in occiput, Mez.

— feeling in small of back, worse on going up-stairs, with an occasional jerking toward hip-joint, Carbon-s.

— in small of back, Ver-a.

**Tetanic,** muscles of back rigid, even tetanic condition may ensue, *Physos.*

**Thighs,** pain in small of back extending to thighs, *Lyc.*

— pains in back and down thighs, *Nit-ac.*

**Thorax,** pain in first dorsal vertebra extending through to thorax and down to lower part of sternum, *Bry.*

**Thrill** of chill creeps up the back, removed by the warmth of stove in the evening, Sul.

— chill in evening with a cold thrill over back, ceasing on lying down, Nitrum.

**Thrill**, cold occasionally over the back with cold sweat on the forehead, anxiety and shuddering, Nat-m.

**Throat**, frequent pressure extending into throat and back, Mag-m.

**Throbbing**, in back in general, *Sil.*, Bar-c., Lyc., *Phos.*, Thuya., Calc-ars., Sep., Alum., Aloe., *Nit-ac.*, *Nat-m.*, Brach.

— and pulsating feeling in back, *Nat-m.*

— in back, worse when coughing, *Nit-ac.*

— isolated in right side of back, Brach.

— in back and grumbling sensation in lower part of small of back, Bar-c.

— and pulsating in back, Phos., Bar-c., *Lyc.*, Thuja.

— aching and beating in back, *Sil.*

— in back a strong pulsating, chiefly while at rest and especially after emotion, Bar-c.

— in back, drives him out of bed at night, Calc-ars.

— in small of back, better when sitting upright, worse when leaning back, Sep.

— in small of back during stool, Alum.

— pain extending up between shoulders, and there becoming a throbbing, Tereb.

**Thrust**, a feeling of a thrust in muscles in small of back when walking, Jatrophia.

**Thumb**, pain in back when sitting still, disappearing on touching, pressure as from a thumb, and tingling in it which increases pain, worse on stooping, drawing upward, *Meny.*

**Tickling**, itching in back, Agar.

**Tightness** in back above hips as if it were a tightness, Mag-c.

— of chest and heaviness in back, Carbo-v.

— of chest with alternating pain in back, Sil.

**Tingling** in back, *Nat-c.*, Arn., Bar-c.

— in back extending to fingers and toes, Sec.

— from back into limbs, with paralysis, *Phos.*

